# Reimagining Residency Training in Syria: A Call for Structured Reform

**DOI:** 10.1055/s-0045-1814097

**Published:** 2025-12-29

**Authors:** Hany Habib, Nour Aloyon Alraqmany, M-Nasan Abdul Baki

**Affiliations:** 1 Department of Internal Medicine, Allegheny Health Network, Pittsburgh, Pennsylvania, United States; 2 Department of Neurology, University of Pittsburgh, Pittsburgh, Pennsylvania, United States; 3 Department of Pathology and Laboratory Medicine, Allegheny General Hospital, Pittsburgh, Pennsylvania, United States

Residency training forms the foundation of medical education, yet in many low- and middle-income countries, including Syria, its educational structure remains fragile. The absence of standardized curricula, limited supervision, and a lack of assessment frameworks hinder the development of clinical reasoning and teaching skills among trainees. Years of conflict and economic hardship have further strained the health care system, leaving most residency programs service-driven rather than learner-centered. Residents often acquire skills primarily through workload and observation, with minimal supervision, structured feedback, or protected educational time.

Competency-based frameworks in high-income countries, such as those endorsed by the Accreditation Council for Graduate Medical Education (ACGME), demonstrate how measurable outcomes, guided supervision, and formative feedback can promote both clinical competence and educational accountability. 1 Adapting these guiding principles can help Syria build its own structured, contextually appropriate, and forward-looking residency training system that reflects local needs, resources, and health care priorities.

International experience shows that improvement is achievable even in resource-constrained settings. In Mozambique, collaboration between Universidade Eduardo Mondlane and the University of California at San Diego introduced competency-based curricula, faculty mentorship, and digital learning access, which improved both training quality and faculty retention. 2 Similarly, the Syrian American Medical Society (SAMS) has implemented continuing medical education and specialty courses across Syria, providing vital training for residents and junior physicians. 3

To advance Syrian residency training, national stakeholders should establish standardized curricula with clear competencies, foster faculty development through “train the trainer” initiatives, and expand access to tele-education and mentorship with international partners. Recognizing residency as structured education rather than a service obligation will not only strengthen clinical training but also enhance the resilience of Syria's health care system.
